# Alteration of Multiple Leukocyte Gene Expression Networks is Linked with Magnetic Resonance Markers of Prognosis After Acute ST-Elevation Myocardial Infarction

**DOI:** 10.1038/srep41705

**Published:** 2017-02-03

**Authors:** A. Teren, H. Kirsten, F. Beutner, M. Scholz, L. M. Holdt, D. Teupser, M. Gutberlet, J. Thiery, G. Schuler, I. Eitel

**Affiliations:** 1 Department of Cardiology/Internal Medicine, Heart Center, University of Leipzig, Germany; 2 LIFE – Leipzig Research Center for Civilization Diseases, University of Leipzig, Germany; 3 Institute of Laboratory Medicine, Clinical Chemistry and Molecular Diagnostics, University of Leipzig, Germany; 4 Institute of Medical Informatics, Statistic and Epidemiology, University of Leipzig, Germany; 5 IZI, Fraunhofer Institute for Cell Therapy and Immunology IZI, Leipzig, Germany; 6 Institute of Laboratory Medicine, University Hospital Munich (LMU) and Ludwig-Maximilian- University Munich, Germany; 7 Department of Diagnostic and Interventional Radiology, Heart Center, University of Leipzig, Germany; 8 University Heart Center Lübeck, University of Lübeck, Medical Clinic II (Cardiology, Angiology and Intensive Care Medicine), Lübeck, Germany

## Abstract

Prognostic relevant pathways of leukocyte involvement in human myocardial ischemic-reperfusion injury are largely unknown. We enrolled 136 patients with ST-elevation myocardial infarction (STEMI) after primary angioplasty within 12 h after onset of symptoms. Following reperfusion, whole blood was collected within a median time interval of 20 h (interquartile range: 15–25 h) for genome-wide gene expression analysis. Subsequent CMR scans were performed using a standard protocol to determine infarct size (IS), area at risk (AAR), myocardial salvage index (MSI) and the extent of late microvascular obstruction (lateMO). We found 398 genes associated with lateMO and two genes with IS. Neither AAR, nor MSI showed significant correlations with gene expression. Genes correlating with lateMO were strongly related to several canonical pathways, including positive regulation of T-cell activation (p = 3.44 × 10^−5^), and regulation of inflammatory response (p = 1.86 × 10^−3^). Network analysis of multiple gene expression alterations associated with larger lateMO identified the following functional consequences: facilitated utilisation and decreased concentration of free fatty acid, repressed cell differentiation, enhanced phagocyte movement, increased cell death, vascular disease and compensatory vasculogenesis. In conclusion, the extent of lateMO after acute, reperfused STEMI correlated with altered activation of multiple genes related to fatty acid utilisation, lymphocyte differentiation, phagocyte mobilisation, cell survival, and vascular dysfunction.

Despite early flow restoration in epicardial coronary arteries, the magnitude of myocardial injury varies substantially in patients with ST-elevation myocardial infarction (STEMI). One of the major determinants of final infarct size and cardiomyocyte death is myocardial reperfusion injury during/after reperfusion of the infarcted vessel^1^. The pathophysiology of reperfusion injury is multifactorial and includes distal embolization/platelet plugging of the microvasculature, release of toxic inflammatory mediators, production of oxygen free radicals, and accumulation of intracellular calcium^2^.

Despite the well-known prognostic relevance of systemic and local inflammatory response for reperfusion injury, data regarding specific molecular markers of the inflammatory response triggered by acute myocardial ischemia are limited. Particularly, leukocyte-driven inflammation plays an essential role in the pathophysiology of reperfusion injury and adverse remodelling in infarcted myocardium^3^,^4^,^5^,^6^. Leukocyte gene expression patterns as assessed by genome-wide transcriptome analysis may therefore provide further insights into the pathophysiology of systemic and microvascular myocardial changes after STEMI with potential diagnostic or even therapeutic relevance.

Cardiovascular magnetic resonance (CMR) has emerged as a promising non-invasive imaging modality for *in*-*vivo* assessment of myocardial damage after STEMI. CMR enables a precise quantification of infarcted and salvaged myocardium, both relevant for the prognosis after STEMI^7^. Furthermore, CMR is able to directly visualise microvascular obstruction (MO), a marker of severe reperfusion injury, which is strongly associated with adverse clinical outcome after STEMI independent from infarct size^8^. However, little is known about the complex molecular processes that associate with the severe myocardial and microvascular tissue damage as visualized by CMR. Therefore, our aim was to identify links between CMR-markers of myocardial damage after acute reperfused STEMI and alterations of the transcriptome on gene- and pathway level in peripheral blood mononuclear cells (PBMC).

## Materials and Methods

### Study population

Patients recruited in this cross sectional trial are participants of the ongoing LIFE-Heart study^9^ admitted for acute STEMI as the first manifestation of coronary artery disease. All participants underwent a full CMR-scan after interventional reperfusion therapy for comprehensive assessment of myocardial damage at day 1–4 after infarction.

The study meets the ethical standards of the Declaration of Helsinki. It has been approved by the Ethics Committee of the Medical Faculty of the University of Leipzig, Germany (Reg. No 276–2005) and is registered by ClinicalTrials.gov (NCT00497887). Written informed consent including agreement with CMR imaging, and genetic analyses has been obtained from all participants enrolled in the study. All methods were carried out in accordance with the relevant guidelines and regulations.

The recruitment phase of the trial was conducted at a single tertiary care centre between August 2008 and November 2010. Patients with infarction undergoing primary percutaneous coronary intervention (PCI) were eligible if the onset of symptoms was less than 12 h before PCI and if they had ST-segment elevation of at least 0.1 mV in ≥2 extremity leads or at least 0.2 mV in ≥2 precordial leads. To ensure that CMR findings reflected acute myocardial injury, patients were not enrolled if they had a previous myocardial infarction (MI). Further exclusion criteria were previous fibrinolysis and patients with contraindications to CMR at study entry such as implanted pacemakers, defibrillators, claustrophobia, or metallic intracranial implants.

### Primary angioplasty and subsequent treatment

Primary PCI was performed according to standard clinical practice. The decision to use bare-metal or drug-eluting stents was left to the discretion of the interventional cardiologist. All patients received 500 mg of aspirin and heparin (60 U/kg body weight) intravenously before PCI. Clopidogrel or Prasugrel (600 mg or 60 mg orally during PCI, if not administered before, followed by 75 mg/day or 10 mg/day for at least 12 months, respectively) was mandatory. Aspirin was given indefinitely at a dose of 100 mg/day. The use of glycoprotein IIb/IIIa inhibitors, angiotensin-converting enzyme inhibitors, beta-blockers, and statins was strongly recommended according to guidelines^10^.

### Angiographic analysis and electrocardiographic analysis

Coronary angiography of the target lesion was performed before and after PCI using standards and projections described elsewhere in detail^7^. For electrocardiographic interpretation, the cumulative ST-segment resolution approximately 90 min after PCI, expressed as the percentage, was calculated by 2 blinded observers as described previously^11^. Categorization was performed in complete (≥70%), partial (<70% to 30%), and no (<30%) ST-segment resolution^2^.

### CMR

CMR was performed on days 1 to 4 after the index event (median time interval of 61 h IQR 44–89 h) using a 1.5-T scanner (Intera CV, Philips Medical Systems, Best, The Netherlands). The well-established infarction protocol includes the assessment of left ventricular (LV) function, mass, volumes, myocardial salvage, infarct size and MO^7^.

For area at risk determination, short-axis slices covering the whole ventricle using a T2-weighted triple inversion recovery breath-hold pulse sequence were obtained using a body coil. Late enhancement images covering the whole ventricle were acquired approximately 15 min after intravenous administration of 0.2 mmol/kg body weight of gadobutrol to assess late MO, and IS (Gadovist, Bayer Schering Pharma, Berlin, Germany). A 3-dimensional inversion recovery turbo gradient echo was used for image acquisition.

### Image analysis

For all quantitative analyses, certified CMR evaluation software was used (cmr42 Circle Cardiovascular Imaging Inc., Calgary, Alberta, Canada). Semi-automated computer-aided threshold detection was used to identify regions of edema, late MO, and infarcted myocardium. A myocardial region was regarded as affected if at least 10 adjacent myocardial pixels revealed a signal intensity of >2 standard deviations (SD) of remote myocardium for edema and >5 SDs in late enhancement images for infarct size (IS)^12^. IS, area at risk, and MO were expressed as percentages of left ventricular mass (% LV), as previously described^7^. If present, MO was included in the overall IS analysis and was also quantified separately. This procedure resulted in following parameters: a) infarct size as percentage of LV mass (IS), b) area at risk as percentage of LV mass (AAR), c) myocardial salvage index (MSI), i.e. AAR-IS/AAR * 100, and d) late MO as percentage of LV mass. For modelling of late MO as function of gene expression levels, we classified patients in the following three groups:

1. patients with no visualised late MO; 2. patients with late MO < MO_median_; 3. patients with late MO ≥ MO_median_. The CMR core laboratory has excellent reproducibility and low interobserver and intraobserver variability for IS and MSI assessment^7^.

### Measurement of gene-expression

In each patient, whole blood was collected (median time interval of 20 h IQR 15–25 h), and further processed to isolate PBMCs for genome-wide gene-expression analysis. Upon immediate centrifugation, samples were stored at 4 °C and transported within 5 hours for PBMC isolation using cell preparation tubes (CPT, Becton Dickinson). Multiple aliquots of PBMCs were stored at −80 °C or liquid nitrogen for further analysis^13^. Total RNA was extracted from PBMC using TRIzol reagent (Invitrogen). 500 ng of RNA (quantified with NanoDrop, Thermo Fisher) were ethanol precipitated applying GlycoBlue (Invitrogen). Gene expression was measured using the HT-12 v4 Expression BeadChip of Illumina (Illumina, San Diego, CA, USA). Wet-lab processing comprised RNA clean-up, precipitation, probe synthesis, hybridisation and scanning of hybridized arrays in accordance to the specification of the manufacturer^14^. Raw data of all 47,323 probes was extracted by Illumina BeadStudio, 47,308 probes could be successfully imputed in all samples. Data was further processed in R/Bioconductor^15^. Individuals with atypical numbers of expressed genes (median ± 3 interquartile ranges (IQR)) were excluded (0.9%). Transcripts not expressed according to Illumina’s cut-off p ≤ 0.05 in at least 50% of all samples were not considered in the analysis. Expression data were quantile normalised and log_2_-transformed^16^. Individuals with atypical expression patterns (quantified as Euclidian distance between expression levels of all individuals and an artificial individual defined as the average of all samples after removing 10% samples farthest away from the average of all samples) were excluded (1.2%, having a distance larger than median + 3 IQR. To further exclude technical artefacts, we defined for each individual a combined score including Illumina’s internal quality control features available for HT-12 v4 quantified as Mahalanobis-distance between all individuals and an artificial individual with average values for these parameters. We excluded atypical individuals with a distance > median + 3 IQR (0.2%). Transcripts were adjusted for known batch effects (Sentrix. ID) applying an empirical Bayes method^17^. Thereby, transcripts still inflated after adjustment according to Bonferronis criteria were excluded. Probes were assigned to genes using Entrez-gene IDs. In consequence, 136 individuals were analysed for 13,483 gene expression probes corresponding to 9,949 unique genes.

### Statistical analysis

Association analysis of late MO as dependent variable with gene expression levels was analysed applying ordered multinomial regression using trichotomized values of late MO as described above. This analysis fully accounts for the non-normal distrubtion of MO values as about 46% of all patients had no visualised late MO, a characteristic that excludes standard linear regression analysis. Association of IS, AAR, and MSI with gene expression levels was done using linear regression. If not stated otherwise, all analyses were adjusted for age, gender, balloon-to-blood time, and balloon-to-CMR time. Thereby, we had 80% power to detect an assocation where gene-expression explains 15%, 18%, and 21% of the investigated variable at a p-value level of 1 × 10^−4^, 1 × 10^−5^, 1 × 10^−6^, respectively. To control multiple testing, we used the false discovery rate (FDR) approach implying default FDR ≤ 5% level of control^18^. This approach has been shown to robustly control for multiple testing in genome-wide gene expression analysis^19^,^20^.

For KEGG-pathway- and gene ontology (GO)-based gene enrichment analysis we performed hypergeometric tests as implemented in the R package “GOstats”^21^,^22^. All genes surviving pre-processing were used in enrichment analysis as background. Network and functional pathway activation analyses was done with QIAGEN’s Ingenuity Pathway Analysis (IPA^®^, QIAGEN Redwood City, www.qiagen.com/ingenuity) using default settings.

## Results

### Patient characteristics

In total, 136 patients fulfilled the inclusion criteria of the study and completed all required laboratory and CMR assessments. The baseline clinical and laboratory characteristics of our study cohort are presented in Tables S1 and S2 in detail including times between pain onset and reperfusion, gene expression measurement as well as CMR. In 54.4% of patients, late MO could be detected by CMR. Patients with no, low, or a high extent of late MO showed significant between-group differences in all remaining prognostic determinants considered in the study (Table 1). Detailed, patients with increased late MO had a higher Gensini score, lower TIMI flow before PCI, lower degree of ST-segment resolution after PCI, and lower ejection fraction in echocardiography. In patients with larger late MO, aldosterone antagonists were more frequently administered (Table S1). In the routine biochemical and haematological analyses, patients with distinct severity of late MO differed significantly for CK-MB, ntproBNP, hsCRP levels, and in white blood cell count (Table S2). No significant between-group differences were observed for relative counts of neutrophils, monocytes, and lymphocytes.

### Overview of associations between PBMC gene-expression and CMR

A summary of associations between adjusted gene expression profiles and considered CMR surrogate markers of clinical outcome is given in Table 2. As anticipated, significant relationship was found between all considered CMR parameters (Supplementary Figure 1).

Both late MO and IS showed relevant relation to gene expression levels whereas no associations at FDR ≤ 5% with AAR and MSI were found. To further investigate this finding, we analysed the distribution of test statistics. This information can be used to estimate the number of genes that are correlated with CMR surrogate markers even when effect sizes of genes are too small to achieve significance at a FDR ≤ 5%. From this analysis we estimate that a large number of genes is associated also with IS, AAR and MSI. Detailed, about a third of all expression probes were related with late MO (eta1 = 35.7%), whereas for the CMR markers MSI, IS, and AAR about a quarter of transcripts were associated, though with smaller effect sizes (Table 2).

### Gene expression changes related to late microvascular obstruction and infarct size

In detail, 398 genes correlated at FDR ≤ 5% with late MO (minimal p – value = 3.51 × 10^−6^, Tables 3 and S3). Upon additional adjustment of these gene associations for smoking, LDL levels, BMI levels, the presence of diabetes mellitus, arterial hypertension, symptom-to-reperfusion time and C-reactive protein, the effect did not change significantly (p – value > 0.2). Functional classification of these genes is summarised in Table S4, respectively. In the subsequent pathway analysis, 202 of these genes (50.6%) could be related to a canonical biological pathways from GO and KEGG, summarised in Table S5. Among central pathways with the highest enrichment were: metabolism (p = 2.21 × 10^−6^), positive regulation of alpha-beta T cell activation (p = 3.44 × 10^−5^), oxidation-reduction process (p = 2.18 × 10^−3^), inflammatory response (p = 4.52 × 10^−3^), antigen processing and presentation of peptide antigen via major histocompatibility complex (MHC) class I (p = 1.69 × 10^−2^), epigenetic regulation of gene expression (2.53 × 10^−2^), response to interferon-gamma (p = 2.67 × 10^−2^), and protein transport (p = 2.80 × 10^−2^).

Association analysis between gene expression levels and IS revealed two significant genes at FDR 5%, *Complement Component* (*3b*/*4b*) *Receptor 1* (*CR1*) and *Interferon Alpha*/*Beta Receptor 1* (*IFNAR1*). Both genes were associated with late MO at FDR values = 2.9% and 6.3%, respectively.

### Prediction of functional downstream effects of associated genes

To further identify functional consequences of changes in gene expression levels associated with different levels of late MO and the other CMR parameters, we performed pathway activation analysis of gene expression networks. Using this approach, we made inferences whether identified enriched pathways are activated or suppressed in the presence of larger late MO levels.

**Figure 1:**
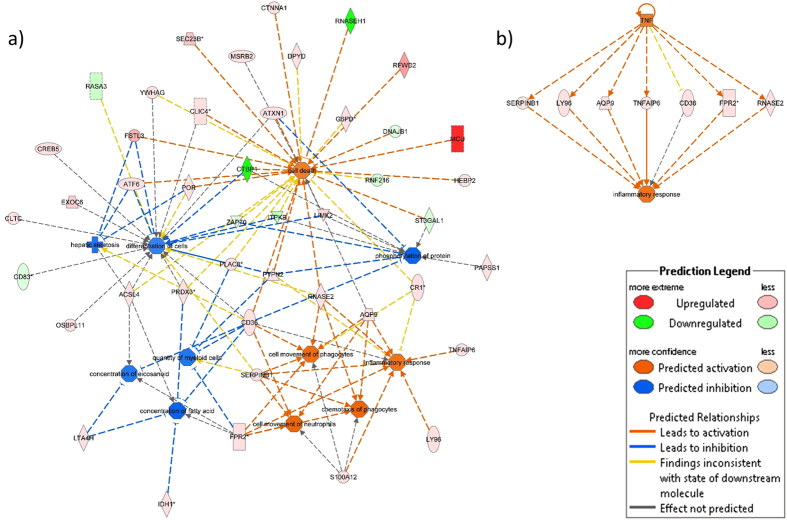
Interaction network of (**a**) top downstream and (**b**) top upstream predicted functional consequences from gene expression level changes of the 100 genes best associated with late MO. “Upregulated” refers to increasing gene expression levels when late MO increased and, vice versa, “Downregulated” refers to decreasing gene expression levels when late MO increased. Stars indicate genes with more than one expression probe - here, gene expression level changes correspond to the strongest associated expression probe. Full names of genes involved in both networks are listed in Table S3.

Firstly, we made prediction for the 100 genes showing the strongest association with late MO (Fig. 1a and b). For more severe late MO, expression patterns of these genes were predicted to result in increased utilisation and subsequent decreased concentration of fatty acid (prediction z-score −1.968; enrichment-p-value = 5.27 × 10^−3^), repressed cell differentiation (z-score −1.480; p = 9.66 × 10^−3^), and decreased myeloid cell count (z-score −1.400; p = 4.87 × 10^−2^). Furthermore, the observed expression changes were predicted to induce the inflammatory response (z-score 1.480; p = 1.92 × 10^−2^), and cell death (z-score 1.480; p = 1.92 × 10^−2^, Fig. 1a).

To identify an upstream factor that might have caused these predicted downstream effects, upstream regulator analysis was additionally performed. Here, tumor necrosis factor (TNF) was suggested to modulate the inflammatory response by upregulating the expression of seven distinct late MO – associated genes (Fig. 1b).

**Figure 2:**
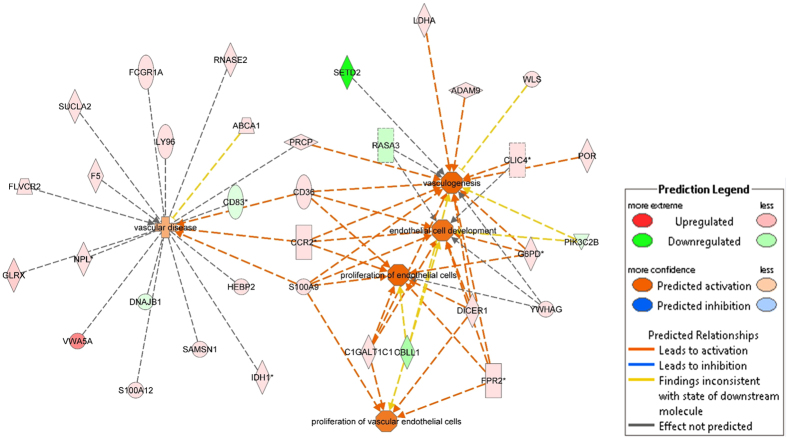
Interaction network of predicted activated or inhibited cardiovascular functions from expression level changes of the 200 genes best associated with late MO. “Upregulated” refers to increasing gene expression levels when late MO increased and, vice versa, “Downregulated” refers to decreasing gene expression levels when late MO increased. Stars indicate genes with more than one expression probe - here, gene expression level changes correspond to the strongest associated expression probe. Full names of genes involved in the network are listed in Table S3.

**Figure 3:**
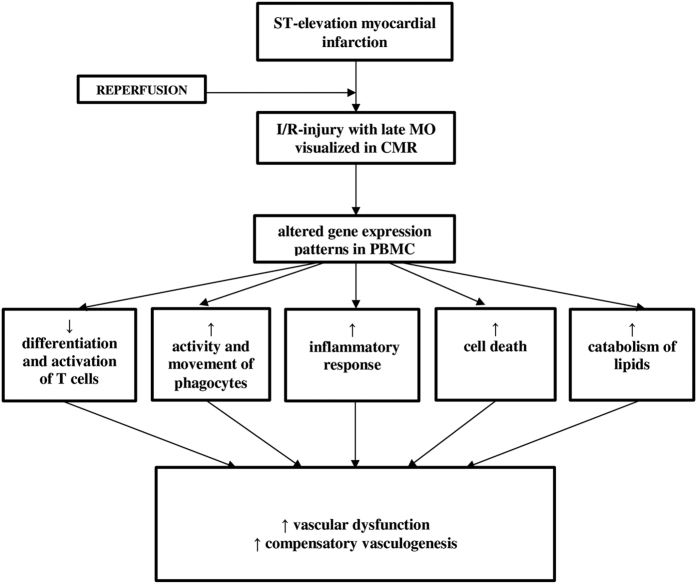
Schematic summary of functional consequences identified by gene expression network analysis related to altered late MO as shown in Figs 1 and 2.

Next, we performed the analogous prediction analysis with focus on phenotypes specific for cardiovascular system, therefore considering the top 200 genes associated with late MO. Multiple links with following processes were identified: promotion of vascular disease (z-score 0.681; p = 2.00 × 10^−2^), increased proliferation of endothelial cells (z-score 2.157; p = 9.53 × 10^−3^), and activation of vasculogenesis (z-score 2.262; p = 1.43 × 10^−2^) (Fig. 2). Cellular and functional consequences of detected gene expression changes PBMC related to lateMO are summarised in Fig. 3.

## Discussion

To our best knowledge, this is the first study characterising global PBMC-specific expression changes in association with established CMR markers of myocardial damage after acute reperfused STEMI. Our sample of 136 detailed phenotyped patients allowed a robust identification of associated genes with moderate effect sizes and related mechanisms. The main findings are: 1. late MO showed strong association with PBMC transcription patterns after STEMI; 2. expression levels associated with larger myocardial damage modulate lipid metabolism, inhibit the differentiation of lymphocytes, promote cell death, and mobilise phagocytes, resulting in inflammation, vascular dysfunction and induction of compensatory vascular repair. Consequently, our data contributes to the understanding of the molecular pathophysiology of mononuclear cells during the post-infarction period suggesting plausible links of the transcriptome to MO, a potent prognostic marker after STEMI.

### Mononuclear cell gene expression patterns and late microvascular obstruction

We found the strongest association between gene expression in mononuclear cells and late MO yielding 398 transcripts correlating with late MO at the level of FDR 5% suggesting multiple candidates for molecular markers of reperfusion injury. (Tables S3 and S4). From the distribution of all p-values, we estimate that in total about 35.7% of all investigated genes are related with late MO (Table 2). Changes in gene expression of PBMC after reperfused STEMI were previously demonstrated in the study of Kiliszek *et al*. comparing 28 STEMI patients with 14 patients having stable coronary artery disease (CAD)^23^. The authors identified 24 relevant genes, from which we also found eight significantly associated at FDR ≤ 5% and 20 at FDR ≤ 20% with late MO. This is considerably more than expected by chance (p_exact_ < 10^−5^ for each of both comparisons) and validates our findings. Another study explored the relationship between peripheral blood gene expression patterns and incident cardiovascular death in 338 patients with suspected or confirmed CAD including 60 (18%) patients admitted for an acute MI. Reported gene expression signature for cardiovascular death was in substantial overlap with expression changes found in patients with acute MI^24^. In the present data, 21 of signature genes reported by Kim *et al*. exhibited the relationship between expression level and late MO. More recently, a set of 559 differentially expressed genes was identified in whole blood of patients after first-time acute MI when contrasting 5 and 22 patients with and without recurrent cardiovascular events, respectively^25^. Our study corroborates pathophysiologic relevance of some of these genes and identifies additional novel genes and possible mechanisms. Concretely, we observed positive correlation of late MO with expression of several genes enhancing lipid synthesis from newly synthetized fatty acids, including c*ytosolic NADP*-*Isocitrate Dehydrogenase* (*IDH1*), and the reported gene *stearoyl*-*CoA desaturase* (*SCD*) involved in synthesis of oleic acid^25^,^26^. Moreover, we found a negative correlation between another reported gene *T*-*cell activation RhoGTPase activating protein* (*TAGAP*) and late MO. *TAGAB* promotes T-cell activation (Table S6). In line with this data, expression changes of many additional genes promoting differentiation and activation of T-cells, and NK-cells, were correlated with reduced late MO, suggesting a protective role of these cells against ischemia - reperfusion (I/R) injury. Exemplarily, our strongest associated gene *WD repeat and SOCS box containing 2* (*WSB2*) was recently shown to downregulate the expression of the *interleukin*-*21 receptor* (*IL21R*)^27^, an important mediator of T-cell and NK-cell differentiation^28^. Patients with more severe late MO showed significantly higher expression of *WSB2* (p = 3.5 × 10^−6^, FDR < 5%, Table 3), and nominally lower expression of *IL21R* (p = 0.01 corresponding to FDR < 9%).

A reperfusion-associated inflammation and microvascular dysfunction after STEMI is promoted by infiltrating neutrophils^29^. Expression levels of potent neutrophil chemotactic factors *N*-*formyl peptide receptor* (*FPR2*), *S100 Calcium Binding Protein A12* (*S100A12*), and *S100 Calcium Binding Protein A9* (*S100A9*) correlated positively with late MO (Fig. 1). Fittingly, *FPR2* antagonists are currently being discussed as promising therapeutics in I/R injury^30^. Expression of both, *S100A12*, and *S100A9*, was previously shown to predict MACE in stable CAD, and acute MI, respectively^31^,^32^. *Chemokine* (*C*-*C Motif*) *Receptor 2* (*CCR2*) that also correlated positively with late MO, is the major receptor for macrophage chemoattractant protein mediating the migration of monocytes at the side of inflammation. In one previous study, targeted silencing of *CCR2* expression ameliorated infarct–associated inflammation and post-infarct left ventricular remodelling in mice^33^.

Distal embolization of plaque debris into small arterioles with subsequent thrombus formation represents a major pathomechanism of severe late MO^34^. Expression of several identified genes is linked with arterial occlusion. This includes examples like *coagulation factor 5* (*proaccelerin*, *F5*), and *carboxyesterase 1* (*CES1*). *CES1* inactivates 80–95% of administered clopidogrel-prodrug in the liver^35^. Therefore, increased myocardial damage among STEMI patients with increased *CES1* expression might result from increased arteriolar thrombotic occlusion, caused by increased hydrolytic degradation of clopidogrel.

Finally, endothelial cell death contributes substantially to the extent of I/R injury^36^. Newly identified genes *mitochondrial calcium uniporter* (*MCU*), *follistatin*-*Like 3* (*Secreted Glycoprotein*) (*FSTL3*), and *LIM Domain Kinase 2* (*LIMK2*) are known to promote cell death and were previously linked to I/R injury. Concordantly, we found increased expression of all three genes among patients with more severe MO, and IS^37^,^38^,^39^, (Figs 1 and 3). In contrast, expression changes promoting endothelial cell proliferation and vasculogenesis were observed (Figs 2 and 3), possibly indicating that counterbalancing mechanisms are initiated but with insufficient activation to prevent functional damage.

### Mononuclear cell gene expression levels and infarct size, myocardial oedema, and myocardial salvage index

In contrast to late MO, we found considerably less association with IS, AAR, and MSI. However, from the total distribution of p-values we estimate that about a quarter of all analysed genes is associated with each of IS, AAR, and MSI, although with effect sizes that are too small to be statistically significant at the FDR 5% level in our dataset. This finding is in line with our observation that frequently similar pathways are effected by altered gene expression in relation to IS, AAR, and MSI (Supplementary Figure 2) Therefore, larger sample sizes may be required for additional analyses for these three parameters.

## Limitations

First, blood samples for microarray analysis were collected early after reperfusion whereas CMR phenotypes were measured up to three days later. Therefore, our analysis is limited to changes of the transcriptome that are relevant for impairments as revealed by CMR that become manifest within days after STEMI. Second, we did not investigate leukocyte gene expression in individuals without STEMI. The principal aim of the study was to identify links between gene expression in leukocytes and established CMR markers of myocardial injury after STEMI, irrespective to the baseline gene expression in unaffected individuals. Third, we found that in depth analysis of transcriptional changes related to IS, AAR, and MSI would requires larger study sample due to the small effect sizes. Therefore, we focussed in our study on gene expression changes and the resulting functional consequences at pathway level that are linked to late MO, where our study was well-powered. Fourth, our results are based on detecting the gene expression on RNA level without considering the translation into the protein. However, recent high throughput proteomic approaches revealed a robust relationship between the gene expression at the RNA and protein level^40^. Finally, although our study confirms known pathway activation states and provides novel hypothesis on genes and pathways linked to more severe STEMI, our analysis is association-based and not yet replicated in an independent setting. Hence, identified relations do not imply causation and should be further validated in future experimental work.

## Conclusions

We found multiple relevant links between gene expression in PBMC and prognostic CMR markers of post-infarction myocardial damage. Several associated genes encode proteins involved in modulation of inflammation, cell survival, and vascular function. For many molecules with previous experimental link to myocardial I/R injury, the present study provides the first corroborating evidence of these links in a clinical setting. Moreover, our analyses revealed several novel molecules and pathways suggesting their involvement in leukocyte-related pathophysiology during post-infarct period. Further studies are warranted to validate the clinical relevance of identified genes and networks for the prediction and improvement of outcome after STEMI.

## Additional Information

**How to cite this article:** Teren, A. *et al*. Alteration of Multiple Leukocyte Gene Expression Networks is Linked with Magnetic Resonance Markers of Prognosis After Acute ST-Elevation Myocardial Infarction. *Sci. Rep.* **7**, 41705; doi: 10.1038/srep41705 (2017).

**Publisher's note:** Springer Nature remains neutral with regard to jurisdictional claims in published maps and institutional affiliations.

## Supplementary Material

Supplementary Information

Supplementary Table S3

## Figures and Tables

**Table 1:** Cardiovascular magnetic resonance characterization of the study sample.

Variable*	All Patients (N = 136)	Late MO absent (N = 62)	Late MO < median (N = 37)	Late MO ≥ median (N = 37)	*p*-value
MR-EF	49.6 (43.6–56.5)	54.2 (48.6–59.7)	47.9 (43.8–52.3)	41.3 (34.9–51.0)	<0.001
AAR (%LV)	33.6 (24.9–49.5)	31.9 (20.3–43.5)	32.5 (26.2–39.1)	48.0 (34.3–56.0)	0.002
IS (%LV)	19.2 (11.1–25.7)	12.3 (3.1–20.4)	19.0 (14.6–22.1)	30.0 (24.7–41.4)	<0.001
MSI (%AAR)	43.8 (27.4–62.3)	58.1 (36.6–87.5)	47.3 (28.5–56.8)	30.0 (10.6–39.5)	<0.001

^*^Values are given as number (%) or median (P25–P75). Late MO – late microvascular obstruction; MR-EF – ejection fraction assessed by magnetic resonance; AAR – area at risk; IS – infarct size; MSI – myocardial salvage index; p-value – result of Kruskal-Wallis test comparing three groups of late MO.

**Table 2:** Summary of association between PBMC gene expression profile and prognostic marker of outcome in patients after reperfused STEMI (n = 136).

Phenotype	Characteristics of gene expression associations			
	min. pval	min. qval	FDR ≤ 5%	eta1
late MO (% LV mass)	3.51 × 10^−6^	0.024	398	35.7%
IS (%LV mass)	2.76 × 10^−6^	0.027	2	27.1%
AAR (% LV mass)	1.44 × 10^−5^	0.125	0	25.4%
MSI	1.59 × 10^−5^	0.150	0	25.6%

^1^min. p:pval minimal p – value, min. q – val: minimal qvalue (i.e. FDR); FDR ≤ 5%: number of associated genes at the default FDR ≤ 5% level of control; estimated percentage of truepositive findings of all 13,486 probes/9,949 genes that could be detected with a very large sample.

**Table 3:** Top 25 transcripts associating with the late MO assessed by CMR at FDR ≤ 5%.

Gene symbol*	Entrez annotated ID	Description	Function according to the gene cards (http://www.genecards.org/)	*p*-value	FDR	OR (95% CI)
*WSB2*	55884	WD Repeat And SOCS Box Containing	modulation of IL21 signal transduction; recognition of substrates for ubiquitin-proteasome- mediated protein degradation	3.51 × 10^−6^	2.36 × 10^−2^	57.03 (10.33–314.91)
*YWHAG*	7532	tyrosine 3-monooxygenase/tryptophan 5-monooxygenase activation protein ɣ	signal transduction by binding phosphoserine or phosphothreonine motif adaptor protein	5.44 × 10^−6^	2.36 × 10^−2^	40.31 (8.19–198.34)
*MCU*	90550	mitochondrial calcium uniporter	mediates calcium uptake into mitochondria	1.10 × 10^−5^	2.57 × 10^−2^	3.57 (25.12–4483.05)
*FSTL3*	10272	follistatin-like 3 (secreted glycoprotein)	binding and antagonizing protein members of the TGF-beta family – 27ctiving, BMP2 and MSTN	1.44 × 10^−5^	2.62 × 10^−2^	102.81 (12.67–834.05)
*LOC728855*	728855	LOC728855	unknown function	1.51 × 10^−5^	2.63 × 10^−2^	0.04 (0.01–0.18)
*DNAJB1*	3337	DnaJ (Hsp40) Homolog, Subfamily B, Member 1	molecular chaperone	2.16 × 10^−5^	2.69 × 10^−2^	0.06 (0.02–0.22)
*MGST1*	4257	microsomal glutathione S-transferase 1	membrane protection against oxidative stress	3.40 × 10^−5^	2.74 × 10^−2^	8.50 (3.09–23.40)
*GOLGA8B*	440270	golgin A8 family, member B	maintaining Golgi structure	3.72 × 10^−5^	2.74 × 10^−2^	0.11 (0.04–0.31)
*SKAP2*	8935	src kinase associated phosphoprotein 2	putatively involved in B-cell and macrophage adhesion processes	3.97 × 10^−5^	2.75 × 10^−2^	19.53 (4.73–80.63)
*RPL26L1*	51121	ribosomal protein L26-like 1	by similarity structural constituent of ribosome	4.94 × 10^−5^	2.77 × 10^−2^	153.20 (13.49–1740.14)
*ATF6*	22926	activating transcription factor 6	activating unfolded protein response target genes during endoplasmic reticulum stress	5.67 × 10^−5^	2.77 × 10^−2^	38.25 (6.49–225.49)
*PLAC8*	51316	placenta-specific 8	regulation of autophagy by facilitating the lysosome-autophagosome fusion	6.93 × 10^−5^	2.78 × 10^−2^	11.13 (3.40–36.50)
*VWA5A*	4013	von Willebrand factor A domain containing 5 A	putatively tumor supressor	7.93 × 10^−5^	2.79 × 10^−2^	158.77 (12.82–1966.87)
*CYFIP1*	23191	cytoplasmic FMR1 interacting protein 1	in protein complex mediates translation repression and actin reorganisation	8.26 × 10^−5^	2.79 × 10^−2^	20.25 (4.53–90.56)
*PGM2*	55276	phosphoglucomutase 2	nucleoside breakdown; interconversion of glucose-1-phosphate and glucose-6-phosphate	8.37 × 10^−5^	2.79 × 10^−2^	40.20 (6.38–253.28)
*TH1L*	51497	Negative Elongation Factor Complex Member C/D	negatively regulates the elongation of transcription by RNA polymerase II	8.79 × 10^−5^	2.79 × 10^−2^	0.01 (0.00–0.10)
*IDH1*	3417	Isocitrate Dehydrogenase 1 (NADP+), Soluble	oxidative decarboxylation of isocitrate to 2-oxoglutarate	8.83 × 10^−5^	2.79 × 10^−2^	14.31 (3.78–54.14)
*ST3GAL1*	6482	ST3 Beta-Galactoside Alpha-2,3-Sialyltransferase 1	catalyzes the transfer of sialic acid to galactose-containing substrates	9.30 × 10^−5^	2.80 × 10^−2^	0.04 (0.01–0.19)
*TMEM167A*	153339	Transmembrane Protein 167 A	early part of secretory pathway	9.36 × 10^−5^	2.80 × 10^−2^	10.32 (3.20–33.29)
*SEC23B*	10483	Sec23 Homolog B (S. Cerevisiae)	protein transport from the endoplasmic reticulum to the Golgi apparatus	9.40 × 10^−5^	2.80 × 10^−2^	78.77 (8.80–704.67)
*CD83*	9308	CD83 Antigen	positive regulation of antigen presentation	9.43 × 10^−5^	2.80 × 10^−2^	0.28 (0.15–0.53)
*MTHFD2*	10797	Methylenetetrahydrofolate Dehydrogenase (NADP + Dependent) 2	methylenetetrahydrofolate dehydrogenase and methenyltetrahydrofolate cyclohydrolase	9.47 × 10^−5^	2.80 × 10^−2^	19.38 (4.37–85.82)
*CD36*	948	CD36 Molecule (Thrombospondin Receptor)	scavenger receptor binds to collagen, thrombospondin, anionic phospholipids, long-chain fatty acids and oxidized LDL	9.50 × 10^−5^	2.80 × 10^−2^	10.10 (3.16–32.25)
*ECRP*	643332	ribonuclease, Rnase A family, 2 (liver, eosinophil-derived neurotoxin) pseudogene	lncRNA of unknown function	9.54 × 10^−5^	2.80 × 10^−2^	3.55 (1.88–6.69)
*ITPKB*	3707	inositol-Trisphosphate 3-Kinase B	modulation of cellular signalling	9.91 × 10^−5^	2.80 × 10^−2^	0.02 (0.00–0.15)

^*^For a full list of all 398 genes with FDR ≤ 5% see Table S3. OR (95% CI) – estimated odds ratio with 95% confidence interval from the ordered multinomial regression model with gene expression as predictor and stratified late MO as response variable.
