# Supplementary material for: Alteration of Multiple Leukocyte Gene Expression Networks is Linked with Magnetic Resonance Markers of Prognosis After Acute ST-Elevation Myocardial Infarction
Source: Sci Rep. 2017 Feb 3;7:41705. doi: 10.1038/srep41705 (PMC5290530; doi:10.1038/srep41705)
Supplement: Supplementary Information [file srep41705-s1.doc]

### SUPPLEMENTARY INFORMATION

**ALTERATION OF MULTIPLE LEUKOCYTE GENE EXPRESSION NETWORKS IS LINKED WITH MAGNETIC RESONANCE MARKERS OF PROGNOSIS AFTER ACUTE ST-ELEVATION MYOCARDIAL INFARCTION**

Teren A., Kirsten H., Beutner F., Scholz M., Holdt LM., Teupser D., Gutberlet M., Thiery J.,

Schuler G., Eitel I.

## Supplementary figures:

### Supplementary Figure 1: Correlation of investigated CMRI measures


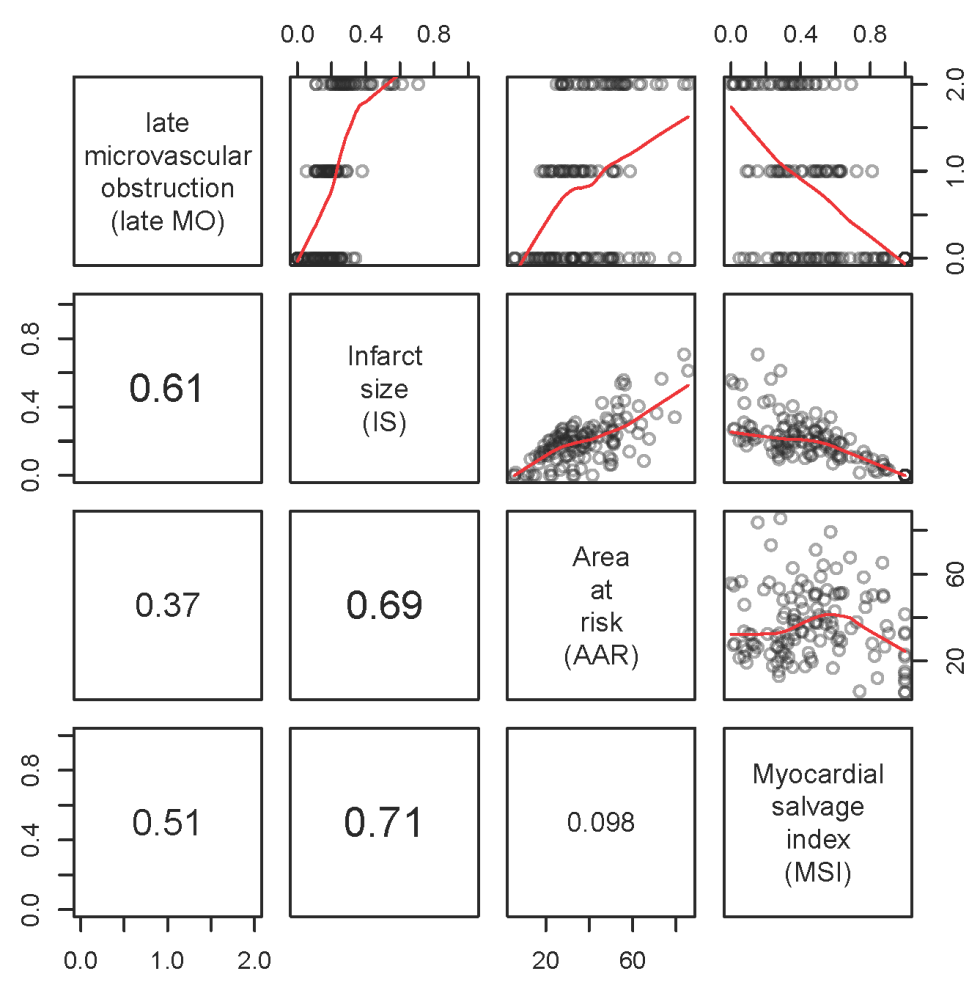


Correlation within the 136 investigated individuals. Numbers represent the correlation r. Red lines indicate a trend as shown by a LOWESS smoother which uses locally-weighted polynomial regression. CMRI – cardiovascular magnetic resonance imaging

### Supplementary Figure 2: Activation patterns of biological processes enriched across investigated CMRI phenotypes.


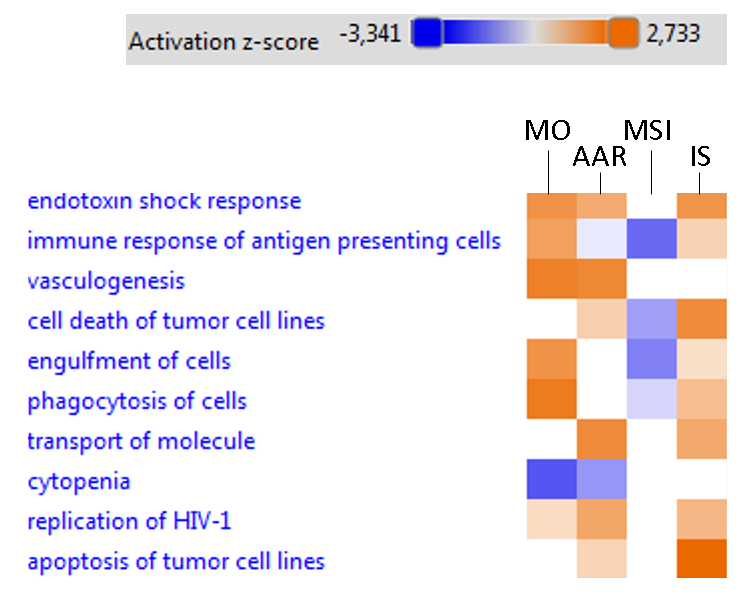


##

## Shown are the top 10 enrichment results found within the top 200 genes of each phenotype. Note that similar patterns, albeit at a different association levels are existent: increased pathway activity is found in relationship to larger myocardial damage i.e. more extensive MO, IS and AAR, and less extensive MSI, respectively. MO – late microvascular obstruction; AAR – area at risk; IS – infarct size; MSI – myocardial salvage index; CMRI – cardiovascular magnetic resonance imaging.

## Supplementary Tables:

### Table S 1 Baseline Characteristics of the study sample

| **Variable** | **All Patients (N = 136)** | **late MO absent (N = 62)** | **late MO < median (N = 37)** | **late MO ≥ median (N = 37)** | ***p*-value** |
| --- | --- | --- | --- | --- | --- |
| Age | 61 (50 - 70) | 67 (50 -72) | 60 (49 - 70) | 56 (50-67) | 0.223 |
| Male sex | 94 ( 69.1) | 38 (61.3) | 29 (78.4) | 27 (73.0) | 0.172 |
| BMI | 28.1 (25.8 - 30.9) | 27.8 (25.4 - 31.0) | 28.7 (26.0 – 31.2) | 27.5 (25.3-29.6) | 0.596 |
| Cardiovascular risk factors |  |  |  |  |  |
| Current smoking | 63 (46.3) | 23 (37.1) | 19 (51.2) | 21 (56.8) | 0.101 |
| Arterial hypertension | 73 (53.7) | 33 (53.2) | 24 (64.9) | 16 (43.2) | 0.213 |
| Hypercholesterolemia | 46 (33.8) | 24 (38.7) | 14 (37.8) | 8 (21.6) | 0.184 |
| Diabetes mellitus | 23 (16.9) | 10 (16.1) | 6 (16.2) | 7 (18.9) | 0.490 |
| CAD severity |  |  |  |  |  |
| Diseased vessels |  |  |  |  | 0.637 |
| 1 | 71 (52.2) | 30 (48.4) | 19 (51.4) | 23 (62.4) |  |
| 2 | 47 (34.6) | 22 (35.5) | 14 (37.8) | 11 (29.7) |  |
| 3 | 17 (12.5) | 10 (16.1) | 4 (10.8) | 3 (8.1) |  |
| Gensini score global | 37.0 (24.0-54.0) | 32 (20-49) | 45 (32-51) | 48 (33-80) | 0.007 |
| Gensini culprit lesion | 32 (17-40) | 24 (16-32) | 32 (24-40) | 48 (32-48) | < 0.001 |
| Anterior MI | 69 (50.7) | 28 (55.4) | 19 (51.4) | 22 (59.5) | 0.345 |
| Killip class  on admission |  |  |  |  | 0.144 |
| 1 | 120 (88.2) | 55 (88.7) | 35 (94.6) | 30 (81.1) |  |
| 2 | 11 (8.1) | 5 (8.1) | 1 (2.7) | 5 (13.5) |  |
| 3 | 2 (1.5) | - | - | 2 (5.4) |  |
| 4 | 3 (2.2) | 2 (3.2) | 1 (2.7) | - |  |
| TIMI flow grade before PCI |  |  |  |  | < 0.001 |
| 0 | 80 (58.8) | 24 (38.7) | 24 (64.9) | 32 (86.5) |  |
| 1 | 8 (5.9) | 6 (9.7) | - | 2 (5.4) |  |
| 2 | 24 (17.6) | 16 (25.8) | 7 (18.9) | 1 (2.7) |  |
| 3 | 24 (17.6) | 16 (25.8) | 6 (16.2) | 2 (5.4) |  |
| TIMI flow grade after PCI |  |  |  |  | 0.187 |
| 0 | 4 (2.9) | - | 1 (2.7) | 3 (8.1) |  |
| 1 | 5 (3.7) | 2 (3.2) | 1 (2.7) | 2 (5.4) |  |
| 2 | 11 (8.1) | 3 (4.8) | 3 (8.1) | 5 (13.5) |  |
| 3 | 116 (85.3) | 57 (91.9) | 32 (86.5) | 27 (73.0) |  |
| Periinterventional  time intervals |  |  |  |  |  |
| Pain to balloon  time (min) | 242 (143-381) | 238 (135-375) | 221 (139-306) | 301 (159-476) | 0.194 |
| Balloon to blood  time (hrs) | 21(15-26) | 19 (16-24) | 22 (16-31) | 21 (15-26) | 0.142 |
| Balloon to CMR  time (hrs) | 61 (44-89) | 69 (47-92) | 53 (41-75) | 56 (39-77) | 0.257 |
| ST-segment  resolution (%) | 54 (20-77) | 65 (31-83) | 45 (21-67) | 50 (3-65) | 0.037 |
| TTE-EF | 50 (45-55) | 52 (47-60) | 50 (45-55) | 50 (40-55) | 0.030 |
| Concomitant therapy |  |  |  |  |  |
| DES implantation | 49 (36.0) | 20 (32.3) | 18 (48.7) | 11 (29.7) | 0.218 |
| Thrombectomy | 44 (32.4) | 20 (32.3) | 11 (29.7) | 13 (35.1) | 0.544 |
| RAAS inhibitors | 135 (99.3) | 61 (98.4) | 37 (100) | 37 (100) | 0.548 |
| Beta-blockers | 135 (99.3) | 61 (98.4) | 37 (100) | 37 (100) | 0.548 |
| Aspirin | 136 (100) | 62 (100) | 37 (100) | 37 (100) | 1.000 |
| Clopidogrel | 116 (77.9) | 51 (82.3) | 29 (78.4) | 26 (70.3) | 0.379 |
| Prasugrel | 30 (22.1) | 11 (17.7) | 8 (21.6) | 11 (29.7) | 0.379 |
| Glycoprotein IIb/IIIa  inhibitor | 136 (100) | 62 (100) | 37 (100) | 37 (100) | 1.000 |
| Statins | 133 (97.8) | 61 (98.4) | 37 (100) | 35 (94.6) | 0.260 |
| Aldosterone antagonist | 12 (8.8) | 3 (4.8) | 1 (2.7) | 8 (21.6) | 0.005 |

* Values are given as number (%) or median (P25 – P75).

BMI – body mass index; CAD – coronary artery disease; MI – myocardila infarction; TIMI –

Thrombolysis in Myocardial Infarction; TTE-EF – ejection fraction determined by trasthoracal

Echocardiography; ST-segment resolution in 90 min. post-PCI; p-value – result of Kruskal-Wallis

test comparing three groups of late MO.

### Table S 2: Baseline laboratory characteristics of the study sample

| **Parameter (units)** | **All Patients (N = 136)** | **lateMO absent (N = 62)** | | **lateMO < median (N = 37)** | **lateMO ≥ median (N = 37)** | | | ***p*-value** |
| --- | --- | --- | --- | --- | --- | --- | --- | --- |
| CK-MB max (µmol/l) | 3.59 (1.77 – 5.92) | 2.08 (0.77-3.56) | | 4.27 (2.14-5.81) | | 6.78 (4.54-9.35) | | < 0.001 |
| CK-MB study inclusion (µmol/l) | 1.57 (0.93 - 2.49) | 1.33 (0.73 - 1.86) | | 1.59 (0.93-2.80) | | 2.23 (1.65-3.53) | | < 0.001 |
| ntproBNP (pg/ml) | 1549 (928 - 2573) | | 1341 (686- 2090) | 1380 (732-2262) | | 1789 (1373 – 2924) | | 0.010 |
| hsCRP (mg/l) | 15.8 (7.8-39.5) | 10.6 (5.4-18.3) | | 26.6 (8.5-56.2) | | 39.6 (15.7-67.1) | | < 0.001 |
| WBC *10^9^/L | 10.4 (8.8-12.7) | 9.5 (8.2-11.9) | | 10.3 (9.4-11.8) | | 11.5 (10.6-14.2) | | < 0.001 |
| Neutrophiles % | 71.5 (66.3-77.2) | 71.7 (66.4-77.7) | | 72.0 (66.8-74.6) | | 72.7 (69.4-78.9) | | 0.156 |
| Monocytes % | 8.6 (7.1-10.1) | 8.3 (7.0-9.7) | | 8.3 (7.1-10.2) | | 8.8 (7.3-10.3) | | 0.529 |
| Lymphocytes % | 18.5 (13.8-23.4) | 18.7 (14.0-23.3) | | 18.2 (14.7-23.0) | | 18.3 (11.6-22.6) | | 0.117 |
| Platelets *10^9^/L | 219 (190-269) | 221 (188-274) | | 217 (192-272) | | 209 (193-232) | | 0.605 |
| * Values are given as median (P25 – P75). CK-MB – creatine kinase – myocardial band  fraction; ntproBNP – N-terminal fragment of brain natriuretic peptide; hsCRP – high  sensitive C-reactive protein; WBC – white blood cell count.  . | | | | | | |  | |

### Table S 3: Full list of 398 genes and corresponding probes significantly associated with late MO at FDR ≤5%

### The table is available as supplemental format (filetype xlsx). OR (95%CI) – estimated odds ratio with 95% confidence interval from the ordered multinomial regression model with gene expression as predictor and stratified late MO as response variable. Gene Definition originates from PANTHER database if not stated otherwise. The strength of associations is expressed by the colouring intensity of respective fields in association column (i.e. reflecting the odds ratio range for late MO). Red colour represents the upregulation, and green colour downregulation of respective gene expression with increasing late MO.

### Table S 4: Functional classification of genes associating with late MO at FDR ≤ 5%

| **Predominant or putative/predicted**  **associated pathway/biological process** | **Gene symbols** |
| --- | --- |
| 1. **METABOLISM** | |
| Glycolysis | *GAPDH, LDHA, PGAM1, PGAM4, PKM2,* |
| Pentose phosphate pathway | *G6PD, PGD, TALDO1* |
| Gluconeogenesis, glycogen metabolism | *FBP1, GYG1, PGM2,* |
| Lipid transport and metabolism | *ABCA1, ACACA, ACOX1, ACOX2, ACSL1, ACSL4, ACSS2, CYB5R1, CYP51A1, GK, HADHB, IDH1, LPCAT3, NUS1, OSBPL11, PLA2G15, PLB1, PLBD1, SCD, SPTLC2* |
| Tricarboxylic acid cycle | *ACSS1, CLYBL, ECHDC1, ME2, SUCLA2* |
| Respiratory chain | *ATP5J2, HIGD1A, NDUFAF1, NDUFV1, SDHB, SDHD* |
| Aminoacid metabolism | *ADI1, BCAT1, GCDH, KYNU, SDSL* |
| Glycoprotein and glycolipid synthesis/stability | *C1GALT1C1, DDOST, GALNT1, GBA, GLT1D1, NPL, OSTC, PIGF, ST3GAL1* |
| Nucleoside metabolism | *DPYD, MTHFD2, NT5C* |
| Sulfat activation/metabolism | *PAPSS1, SQRDL, SUMF1* |
| Drug/xenobiotics metabolism | *CES1, GSTO1* |
| Calcium homeostasis | *FAM82A2, FLVCR2, MCU* |
| Redox homeostasis | *GLRX, GPX1, MGST1, MSRB2, PRDX3, TXN, TXNDC17, TXNL1* |
| 1. **REGULATION OF NUCLEAR GENE EXPRESSION** | |
| Epigenetic regulation of transcription, independent  from RNA turnover | *BRD9, CBX7, H3F3A, HABP4, KANSL3, MPHOSPH8, SCMH1, SETD2, ZNF493* |
| microRNA regulation and turnover | *DICER1, ZCCHC11* |
| Long non-coding RNA/pseudogene | *ANXA2P1, DSTNP2, ECRP, LOC100132707, LOC339192, LOC728855, OIP5-AS1, UQCRHL* |
| Translation regulation | *FARSA, SECISBP2* |
| Ribosomal biogenesis | *CHD7, DDX47, RPL23A, RPL5, SNORA24, SNORA62, SNORA80, TSR2, UTP23* |
| RNA processing/trafficking/degradation | *AGFG1, CDC40, HNRNPU, MVP, NCBP1, NUP205, NXT2, RNASE2, RNASE3, RNASEH1, RTCD1, SIDT1, SNRPA1, SUGP2,* |
| Receptor-mediated transcription regulation | *CREB5* |
| Transcriptional repression | *AES, C11orf74, KLF12, TH1L, ZBTB34* |
| Transcriptional activation | *GCN1L1, RFX5, TCEA2, ZNF211, ZNF275, ZNF837* |
| 1. **MODULATION OF IMMUNE RESPONSE** | |
| Anti-inflammatory | *ADNP, CNOT1, COMMD10, LDOC1, LILRB4, MAP4K1, PTPN2, SETD6* |
| Pro-inflammatory | *AKIRIN, CARD16, CD93, FCGR1A, FCGR1B, GLIPR1, IFNG, LTA4H, LY96, MMP25, MNDA, MTDH, NLRC4, S100A12, S100A9, SIRPA, TLR1* |
| Complement inhibition | *CD55, CR1* |
| Chemokine activity | *CCR2, FPR2, QPCT* |
| Modulation of NK-cell function/development | *MLL5, NKTR* |
| Modulation of T-cell function/development | *CD247, CD6, CD83, ITFG1, ITPKB, LGALS1, LY9, PIK3C2B, PRKCQ, PRKCZ, SKAP1, SYTL1, TAGAP, UBASH3B, ZAP70, ZBTB25* |
| Modulation of B-cell function/development | *AKNA, FANCD2, PLEKHA1, SAMSN1, SKAP2* |
| 1. **TRANSMEMBRANE/INTRACELLULAR TRANSPORT** | |
| Transmembrane transporter | *AQP9, SLC22A4, SLC25A37, SLC25A42, SLC26A8, SLC44A1, SLC9A9, TMCO3* |
| Scavenger receptor | *CD163, CD36, CLEC4E, STAB1* |
| Phagocytosis | *RAB31, RAB32, TM9SF2, TM9SF3* |
| Exocytosis | *EXOC6, RTN3,* |
| Endocytosis/Intracellular vesicle trafficking | *AFTPH , ARF4, ARL8B, ARRDC4, CLTC, COPA, FCHO2, IFT20, NSF, PLIN3, RAB10, RAB18, RAB6A, RPS6KC1, SAR1B, SDCBP, SEC23B, SEC24A, SFT2D1, SH3GLB1, SNX27, SNX3, TOM1L2, TRAPPC6A* |
| 1. **REGULATION OF CELL CYCLE, SURVIVAL, ADHESION, AND MOTILITY** | |
| Cell proliferation | *ANAPC4, CDC25B, CDK5RAP3, CREG1, FAM53B, MCTS1, MIS12, POGLUT1, RGCC, SNRK, TSPYL2* |
| Hematopoiesis | *IL11RA* |
| Angiogenesis | *GLMN* |
| Cell survival/apoptosis | *BOK, DRAM1, FAM105A, HBXIP, HEBP2, PLAC8, PMAIP1, SLFN11, VMP1* |
| Cell-cell/cell-ECM communication/adhesion | *ADAM9, CTNNA1, CYFIP2, FAM129B, LGALS8, NPTN, PANX2, PVRL3, TNFAIP6, VCAN* |
| Cytoskeleton organization/cell motility | *ARPC5, CORO1C, CYFIP1, EVL, GMFG, LIMK2, MPP1, MYL6, MYL6B, RHOQ,* |
| 1. **SUBCELLULAR COMPARTMENT INTEGRITY** | |
| Golgi structure/function | *CTBP1, GOLGA8B, GPR89A, YIPF4* |
| ER structure/function | *ATF6, DNAJB1, ERLIN2, JKAMP, POR, WRB* |
| Sarcolemma integrity | *DYSF* |
| Mitochondrial structure/function | *DHX30, MINOS1, MRPL35, MRPS23, TIMM21* |
| 1. **PROTEIN STABILITY AND TURNOVER** | |
| Chaperone/Adaptor | *CCDC136, DNAJC13, DOK3, PDCL3, PDLIM7, SH3KBP1, YWHAG* |
| Protein folding/sorting | *ATP6V1A, ATP6V1D, CALU* |
| Ubiquitine degradation | *CBLL1, CNPY2, KLHL2, POMP, PSMA1, PSMA6, RFWD2, RNF181, RNF216, UEVLD, WSB1, WSB2* |
| Peptidase activity | *CPD, CTRC, LXN, PRCP, SERPINB1, SERPINB8, TIMP1, VWA5A* |
| 1. **SPECIFIC CHEMICAL BINDING – DEPENDENT REGULATORY ACTIVITY** | |
| GTPase activation | *AGAP6, ARHGAP33, RABGGTB, RASA3, TBC1D10C* |
| GTPase nucleotide exchange modulation | *CCPG1, DENND2D, DOCK5, LAMTOR2, RALGDS* |
| G-protein coupled receptor signalling | *GNG10* |
| Coagulation cascade | *F5* |
| Calcium-dependent binding/transport | *ANXA2, BEST1, FBLN2, GCA, STRN4, WDR74,* |
| 1. **OTHER/ UNKNOWN** | |
| Other | *ACPP, ATXN1, MCEMP1,C1orf43, C5orf39, C6orf211, CLIC4, DMWD, FSTL3, METTL7A, MS4A4A, OVGP1, PLP2, PPP2CA, RBMS1, RDH11, RPAIN, RPL26L1, SBK1, SCCPDH, SLITRK4, SMPDL3A, TCN2, TDRD9, TMEM2, WLS, ZMYND19, SNX29* |
| Unknown | *ANKRD33, ANKRD36C, C19orf6, CEP85L, CYSTM1, ECHDC2, EVI2A, FAM178A, FAM198B, FAM20A, FAM72D, FAM89A, FBRSL1, FOXP1-IT1, KIAA0182, KIAA1683, KLHDC4, KTI12, LMBR1, LOC100129697, LOC100507369, LOC729558, MOSPD2, MSANTD2, NPIPL2, NUCKS1, ODF2L, RBM18, RBM47, TMEM144, TMEM165, TMEM167A, TMEM203, TTC13, UHRF1BP1L, YIF1B, ZFP106, ZUFSP* |

* Genes are shown with FDR ≤5% level, functional classification was performed according to Gene

Cards - related references (http://www.genecards.org/). Genes within each biological theme and

category are ordered alphabetically. Green = negative; Red = positive correlation with late MO.

***Table S 5***: GO- and KEGG - Gene set enrichment analysis of genes associating with late MO after STEMI at FDR ≤ 5%

| **Name of the Pathway** | **Pathway**  **size** | **Pathway ID** | **Gene enrichment set** | ***p - value*** |
| --- | --- | --- | --- | --- |
| NADPH regeneration | 13 | GO:0006740 | *G6PD, IDH1, PGAM1, PGD, TALDO1* | 1.06x10^-6^ |
| Metabolic pathways | 1035 | KEGG:01100 | *ACACA, ACOX2, ACSL4, ACSS2, ADI1, ATP5J2, ATP6V1A, C1GALT1C1, CYP51A1, DPYD, FBP1, G6PD, GAPDH, GBA, GCDH, GK, IDH1, ITPKB, KYNU, MTHFD2, NDUFV1, PAPSS1, PGAM1, PGAM4, PGD, PIGF, PIK3C2B, PLB1, RDH11, SDHD, SPTLC2, SUCLA2, TALDO1, UQCRHL* | 2.21x10^-6^ |
| positive regulation of alpha-beta T cell activation | 40 | GO:0046635 | *CCR2, CD55, CD83, ITPKB, PRKCQ, PRKCZ* | 3.44x10^-5^ |
| glucose catabolic process | 75 | GO:0006007 | *FBP1, G6PD, GAPDH, PGAM1, PGAM4, PGD, TALDO1* | 1.72x10^-4^ |
| positive regulation of NF-kappaB transcription factor activity | 113 | GO:0051092 | *MTDH, NLRC4, PRDX3, PRKCQ, PRKCZ, PSMA6, S100A12, S100A9* | 4.05x10^-4^ |
| regulation of inflammatory response | 211 | GO:0050727 | *CCR2, CD55, CR1, GBA, GPX1, PSMA6, PTPN2, S100A12, S100A9, SETD6* | 1.86x10^-3^ |
| oxidation-reduction process | 508 | GO:0055114 | *ACACA, ACOX2, ACSS2, ATP5J2, GCDH, GNG10, GPX1, GYG1, HIGD1A, NDUFAF1, NDUFV1, POR, SDHD, SQRDL, STAB1, SUCLA2, TXNDC17* | 2.18x10^-3^ |
| response to external stimulus | 1725 | GO:0009605 | *ACSL4, AES, AKIRIN2, BEST1, CCR2, CD36, CD55, CR1, DICER1, FPR2, G6PD, GBA, GPX1, HABP4, HBXIP, IFT20, KYNU, LXN, LY96, MPP1, MTDH, NLRC4, PLAC8, PLB1, PLP2, POR, PRDX3, PRKCQ, PSMA6, PTPN2, RDH11, RNASE2, S100A12, S100A9, SDCBP, SETD6, SLFN11, STAB1, TLR1, UBASH3B, ZCCHC11* | 4.15x10^-3^ |
| **Name of the Pathway** | **Pathway**  **size** | **Pathway ID** | **Gene enrichment set** | ***p - value*** |
| inflammatory response | 287 | GO:0006954 | *CD163, FPR2, LXN, LY96, MMP25, NLRC4, PRKCQ, PRKCZ, STAB1, TLR1, TNFAIP6* | 4.52x10^-3^ |
| regulation of cell-cell adhesion | 82 | GO:0022407 | *FSTL3, LGALS1, PRKCQ, RGCC, UBASH3B* | 9.35x10^-3^ |
| leukocyte activation | 562 | GO:0045321 | *CCR2, CD55, CD83, CD93, CHD7, GLMN, ITPKB, LGALS1, MNDA, PRKCQ, PRKCZ, PTPN2, S100A12, SKAP2, SNX27, TLR1* | 1.59x10^-2^ |
| antigen processing and presentation of peptide antigen via MHC class I | 95 | GO:0002474 | *CD36, FCGR1A, FCGR1B, PSMA6, SAR1B* | 1.69x10^-2^ |
| regulation of gene expression, epigenetic | 143 | GO:0040029 | *DICER1, GLMN, GPX1, MPHOSPH8, TDRD9, ZCCHC11* | 2.53x10^-2^ |
| response to interferon-gamma | 107 | GO:0034341 | *FCGR1A, FCGR1B, GAPDH, KYNU, PTPN2* | 2.67x10^-2^ |
| protein transport | 1279 | GO:0015031 | *AFTPH, ARF4, ATP6V1A, CD36, CLEC4E, COPA, EXOC6, GLMN, MVP, NLRC4, NSF, NUP205, PRKCZ, RAB18, RAB31, RAB6A, RGCC, RPAIN, RPL5, S100A12, SAR1B, SDCBP, SEC23B, SNX27, SYTL1, TIMM21, TLR1, UEVLD, YWHAG* | 2.80x10^-2^ |

* All genes associating at FDR ≤ 5% with late MO were included in the analysis. Gene symbols

within each set are ordered alphabetically.
